# Clinical analysis of EBRT vs TLM in the treatment of early (T1-T2N0) glottic laryngeal cancer

**DOI:** 10.7150/jca.46487

**Published:** 2020-09-23

**Authors:** Jing Shen, Ke Hu, Jiabin Ma, Hongnan Zhen, Hui Guan, Wenhui Wang, Fuquan Zhang

**Affiliations:** Chinese Academy of Medical Sciences & Peking Union Medical College, NO.1 Shuaifuyuan Wangfujing, Dongcheng District, Beijing 100730, People's Republic of China

**Keywords:** glottic laryngeal cancer, external beam radiation therapy, transoral laser microsurgery, voice outcome, VHI

## Abstract

**Objective:** To analyze the clinical efficacy of external beam radiation therapy (EBRT) vs transoral laser microsurgery (TLM) in patients with early glottic laryngeal carcinoma (T1-T2N0) and the effect of treatment choice on vocal function.

**Methods:** A retrospective analysis of patients with T1-T2N0 glottic laryngeal carcinoma who underwent EBRT or TLM between January 2012 and December 2018 in PUMCH. The Kaplan-Meier method was used to analyze local control, progression-free survival and overall survival, and the VHI-30 scale was used to evaluate the effects of EBRT and TLM on vocal function.

**Results:** A total of 185 patients, all with pathologically confirmed squamous cell carcinoma, were enrolled. The median age was 62 years (38-88). N0 disease was confirmed by imaging: 142/185 (76.76%) patients had T1N0 disease, and 43/185 patients (23.24%) had T2/N0 disease. A total of 91/195 (49.19%) patients received an EBRT dose of 66-70 Gy/30-35f, at 2.0-2.3 Gy/f. 94/185 (50.81%) patients received TLM. The median follow-up time was 42 months (12-92), and the 3-year LC, PFS, and OS rates for the EBRT and TLM groups were 96.9% vs 94.1%(p=0.750), 95.3% vs 93.1%(p=0.993) and 93.3% vs 95.4%(p=0.467), respectively. The VHI-30 scales were used at the baseline showed no significant difference between the two groups 19.20±3.324 vs 21.65±9.80 (p=0.250), but the EBRT group had a low voice handicap after treatment, 10.24±6.093 vs 19.45±5.112 (p=0.001) (6 months) and 9.45±5.112 vs 14.97±7.741 (12 months). No CTCAE grade 3 or above side effects were observed in the EBRT group, but 3 cases of vocal cord stenosis were observed in the TLM group.

**Conclusion:** The application of EBRT for early glottic laryngeal carcinoma (T1-T2N0) had an obvious curative effect with high LC and OS rates, no serious side effects, and a low voice handicap rate.

## Introduction

For patients with early glottic laryngeal cancer (T1-T2N0), local treatment, including total/half laryngectomy, transoral laser microsurgery (TLM), and external beam radiation therapy (EBRT), is usually recommended because the probability of lymph node metastasis is 0% ~2%^1^. In patients with early glottic laryngeal cancer after total/partial laryngectomy, the 5-year local control (LC) rate can exceed 90% but with a high voice handicap. To improve the quality of life of patients with early glottic laryngeal cancer, we use other treatments, such as TLM or EBRT, to preserve or maintain the patient's vocal function. Related research has reported that the 5-year LC rate for TLM is approximately 75.81~93.8% for patients with early glottic laryngeal cancer^2^-^4^. EBRT is associated with a similar or even better survival outcome, and the 5-year LC rate is approximately 85~95% for patients with T1N0 disease 5, 6. Although the preferred treatment for early glottic laryngeal cancer is still controversial in terms of the therapeutic effect on the tumor, the goals of treatment are to preserve the vocal function of the larynx and aim for the best voice outcome.

In contrast to the effects of TLM on vocal function, the effects of EBRT on the vocal function of patients with early glottic laryngeal cancer are still controversial 7-9. Therefore, we applied the Voice Handicap Index-30 (VHI-30) scale to evaluate the effect of EBRT and TLM on vocal function. The VHI-30 scale is a self-assessment tool for patients that includes functional, physiological and psychological components. It is widely clinically used. The lower the score is, the better the vocal function will be 10, 11.

The aim of this study was to analyze the efficacy of EBRT and TLM in patients with early glottic laryngeal carcinoma (T1-T2N0), the relevant influencing factors, and the use of the VHI-30 scale to evaluate the effect of EBRT and TLM on voice quality.

## Methods and materials

### Patients

A nonrandomized, longitudinal retrospective analysis was conducted at the Department of Radiation Oncology, Peking Union Medical College Hospital, from January 2012 to December 2018. The inclusion criteria were as follows: patients with T1N0 and limited T2N0 glottic laryngeal cancer; microscopic examination- and biopsy-confirmed pathological squamous cell carcinoma throughout the mouth and throat; an evident parapharyngeal space and prepharyngeal gap on imaging; and N0 disease. Voice outcome and perceptual voice analyses were conducted using the VHI-30, and the data were collected at the baseline of the EBRT or TLM treatment and at various time points during follow-up at 6 months and 12 months.

### Treatments

The patients were separated into two groups for analysis based on treatment strategy. The treatment group consisted of individuals treated with EBRT and the control group consisted of individuals treated with TLM. The details of two treatment strategy were described below.

### EBRT

All patients underwent CT simulation in the supine position (16 rows - Philips Bril-Liance CT Bigbore, Deventer, Netherlands) with head and neck shoulder positioning. The total tumor volume, gross tumor volume (GTV) and CTV (clinical tumor volume) were plotted on the axial CT image. GTV sketching was based on MRI findings. EBRT treatment was administered using a 6-MV X-ray apparatus. For standard fraction (SF) radiotherapy, three-dimensional conformal radiation therapy (3D-CRT) was administered, and for accelerated fraction (AF) radiotherapy, fixed-field intensity-modulated radiation therapy (FF-IMRT), volumetric intensity-modulated radiotherapy (VMAT) or Hi-Art spiral tomography therapy (TOMO) was performed. The CTV included the whole throat area; the GTV included the laryngeal area. The CTV and GTV were evenly placed 5 mm apart to form the PCTV and PGTV. The doses administered were as follows: PCTV, 60-70 Gy/30-35 f (2 Gy/f) and PGTV, 66-69 Gy/30 f (2.2-2.3 Gy/f). The radiation therapy program was generated on an Eclipse or a tomographic treatment planning system with the goal of providing at least 95% of the prescribed dose to 95% of the PCTV and PGTV. The organs at risk (OARs) were limited to the spinal cord, at 0.1 cc <45 Gy. To ensure the accuracy of the treatment, patients receiving TOMO underwent MVCT and online registration every day; patients undergoing FF-IMRT or VMAT received weekly CBCT guidance and were matched online with an error of 3 mm.

### TLM

Transoral CO2 laser microsurgery was carried out under general anesthesia using a Sharpen laser with a digital accolade micromanipulator typically set in continuous or continuous superpulse mode. In most cases, the tumor was first transected to assess the depth of tumor invasion and then resected in two pieces. Resections varied from type I to type V of the European Laryngological Society (ELS) class action as during the study period, tumors requiring larger resections were treated by RT according to Dutch guidelines. Tumor specimens were pinned on a piece of cork and were sent, along with descriptive drawings, to the pathologist for histological examination. In most cases, standard practice was followed, and additional wound bed biopsies were taken and contained separately. Typically, wound bed biopsies were taken at five different points: four were taken at the edges of the tumor ground, and one deep biopsy was taken in the middle of the tumor ground. If the surgeons found it necessary, extra biopsies could be taken.

### Evaluation of vocal function

The Chinese version of the VHI is a validated 30-item questionnaire. The VHI-30 is a widely used voice evaluation scale comprising a total of 30 questions that address function, physiology, and emotion. Each part includes 10 questions relating to factors including hoarseness, difficulty breathing, difficulty with pronunciation, and vocal cord distortion. Patients score each item by selecting a response based on self-evaluation from a five-point Likert scale ranging from 0 to 4, as follows: never occurs (0 points), almost never occurs (1 point), sometimes occurs (2 points), almost always occurs (3 points), always occurs (4 points). The sum of the scores constitutes the total VHI-30 score, which ranges from 0 to 120. A higher score indicates a worse voice-related outcome^10^**.**

### Observation indicators and side effects

The baseline indicators of the patients were collected before radiotherapy, and the patients were followed up every 3 months for 2 years and every 6 months for 2 to 5 years after EBRT. The LC rate was the time interval from the end of treatment to the occurrence of local or regional recurrence. Progression-free survival (PFS) was defined as the time to local or regional recurrence or distant metastasis during or after treatment, and all time intervals were based on the time between the first event and death due to the disease. Overall survival (OS) was defined as the time from diagnosis to death or last follow-up. If no relevant event occurred, PFS or OS was defined from diagnosis to the last follow-up visit.

### Statistical analysis

Statistical analysis was performed using SPSS version 25.0. The chi-square test was used to classify variables. The normality of continuous variables was determined using the Kolmogorov-Smirnov method. Student's t-test was used to evaluate variables with normal distributions, and the Mann-Whitney U test was used for variables with non-normal distributions. Data are expressed as the mean±SD. The Kaplan-Meier method was used to estimate the OS, DFS and LC rates, and the significance of prognostic factors for survival was evaluated by a univariate log-rank test. Multivariate analysis of the covariates selected in the univariate analysis was performed using Cox proportional regression. A p value <0.05 was considered statistically significant.

## Results

### Patient data

A total of 185 patients were enrolled, as shown in Table 1. All patients had a KPS ≥ 80 points and a median age of 62 years (38-88 years); 95.14% (176/185) of the patients were male, and 48.65% (90/185) of the patients had a smoking index ≥ 400. All patients had pathologically confirmed squamous cell carcinoma, and imaging examinations indicated N0 disease. According to the AJCC 7^th^ edition staging criteria, 76.76% (142/185) of the patients had T1N0 disease, of which 65.50% (93/142) had T1a disease, 34.50% (49/142) had T1b disease, and 23.24% (43/185) had T2N0 disease. A total of 65.95% (122/185) of the patients had well-differentiated squamous cell carcinomas, 28.65% (53/185) had moderately differentiated cancers, and 5.4% (10/185) had poorly differentiated cancers. Ninety-one patients (49.19%) received EBRT, and ninety-four patients (50.81%) received TLM treatment. See Table 1 for details.

### Clinical efficacy analysis

**Figure 1:**
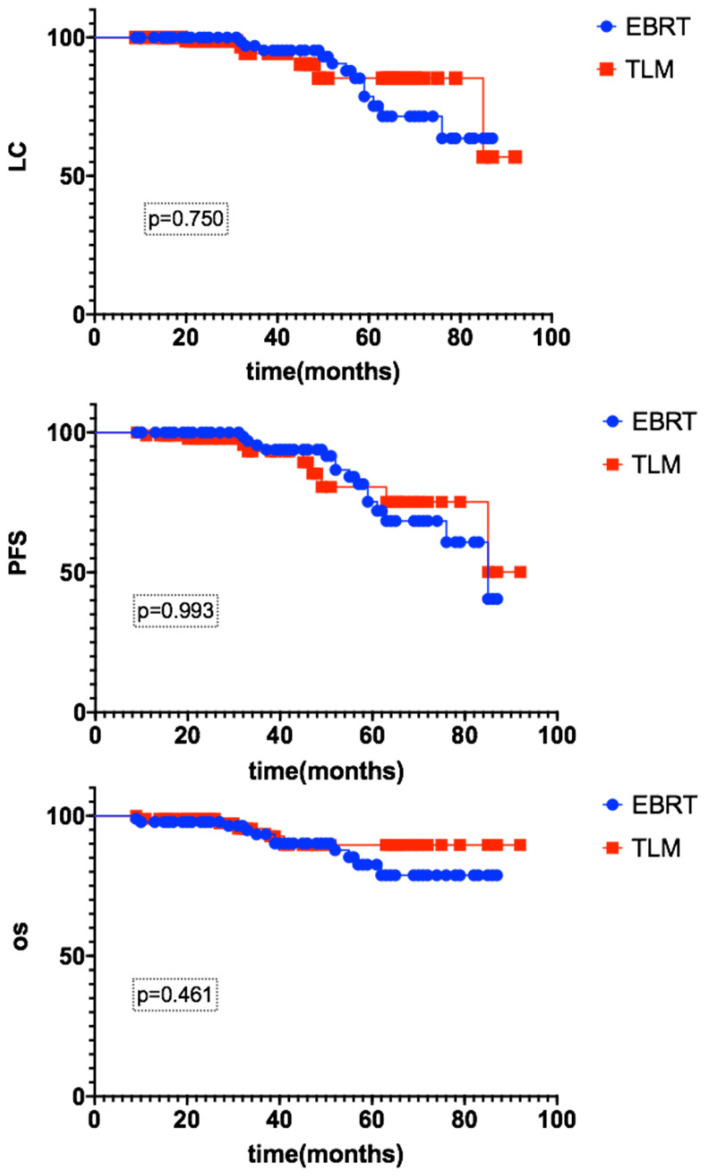
The 3-year LC, PFS and OS rates of early glottic carcinoma patients after EBRT or TLM

The median follow-up time was 42 months (12-92). For the EBRT group, the 3-year LC rate was 96.9%, and the PFS and OS rates were 95.3% and 93.3%, respectively. For the TLM group, the 3-year LC rate was 94.1%, and the PFS and OS rates were 93.1% and 95.4%, respectively. There were no significant differences between the EBRT and TLM groups, as shown in Figure 1.

### Subgroup analysis

**Figure 2:**
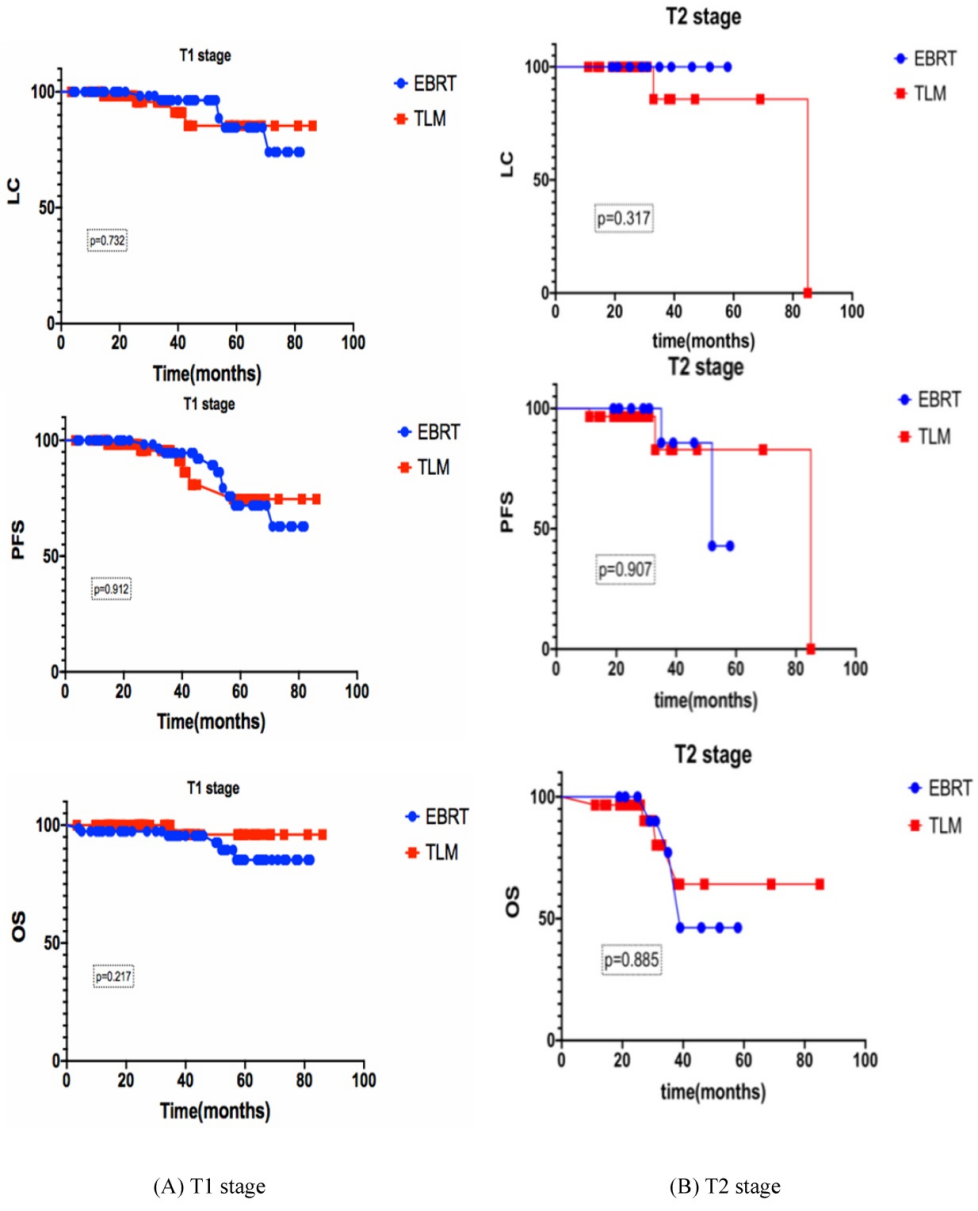
Subgroup analyses of the 3-year LC, PFS and OS rates of early glottic carcinoma patients after EBRT or TLM. Subgroup analyses of the 3-year LC, PFS and OS, (A) stand for T1 stage, (B) stand for T2 stage, there is no significant difference between EBRT and TLM in both stages.

For stage T1 (n=142), the 3-year LC, PFS and OS rates of the EBRT group (n=78) and the TLM group (n=64) were 96.4% vs 95.6% (p=0.732), 94.8% vs 91.1% (p=0.912), 95.6% and 96.0% (p=0.217) respectively. For stage T2 (n=43), the 3-year LC, PFS and OS rates of the EBRT group (n=13) and the TLM group (n=30) were 100% vs 85.7% (p=0.317), 83.3% vs 82.9% (p=0.907), 75% and 80.2% (p=0.885) respectively, as shown in Figure 2.

### Side effects

For the EBRT group (n=91), a total of 36 out of 91 (39.6%) patients had grade 1 acute skin side effects, 54 out of 91 (59.3%) patients had grade 2 acute skin side effects, and 1 out of 91 (1.1%) patients had grade 3 acute skin side effects. Patients with grade 1 laryngitis for 22/91 (24.2%) patients, and those with grade 2 laryngitis accounted for 69/91 (75.8%) patients. In terms of chronic side effects, grade 1 skin reactions occurred in 26/91 (28.57%) patients, and grade 1 edema occurred in 16/91 (17.58%) patients. No grade 3 or higher toxic side effects were observed.

For the TLM group (n=94), 1 patient suffered from asphyxia and hypoxia leading to a vegetative state, 3 patients suffered from massive hemorrhage and underwent reoperation, and 3 patients with chronic side effects underwent surgical treatment for vocal cord stenosis.

### Voice outcome

**Figure 3:**
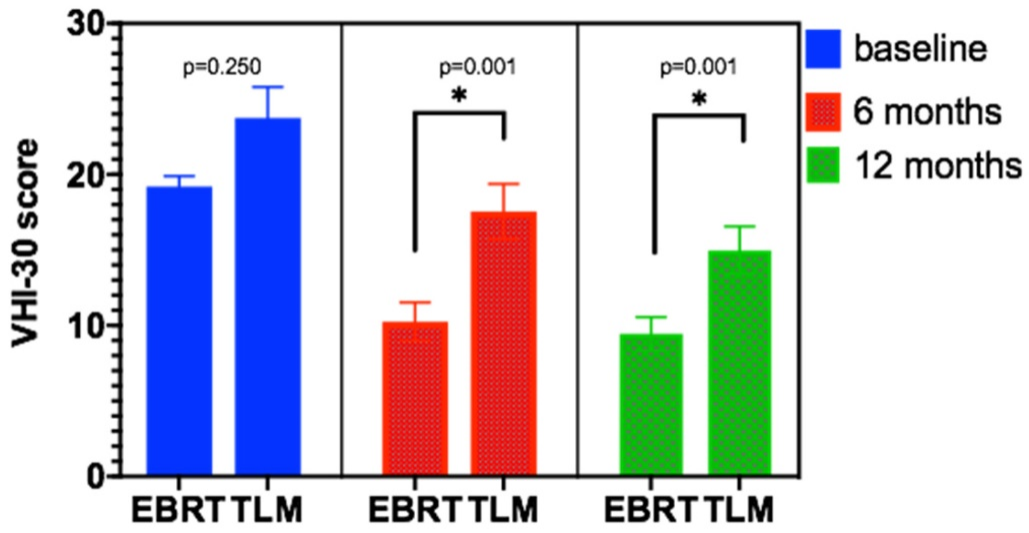
VHI-30 score at baseline and after EBRT or TLM

The VHI-30 scale was used to analyze the vocal function of patients at baseline (before treatment) and at 6 months and 12 months after EBRT and TLM treatment. The results for the EBRT group were 19.20±3.324, 10.24±6.093, and 9.45±5.112, respectively. For the TLM group, the results were 21.65±9.80, 17.54±8.913, and 14.97±7.741, respectively. There was no significant difference in baseline levels between the two groups, but there was a significant difference in vocal quality between the EBRT group and TLM group at 6 months and 12 months after treatment (p < 0.01). See Table 3 and Figure 3 for details.

### Failure mode and follow-up treatment

Local recurrence occurred in 23 patients: 7 patients in stage T1aN0, 12 patients in stage T1bN0, and 4 patients in stage T2N0. The average time from treatment to recurrence was 17 months (3-45 months), and 18 out of 23 (78.26%) patients experienced recurrence within 2 years after treatment. A total of 18 out of 23 (78.26%) of the recurrence sites were in the vocal cord zone, and 6 out of 23 patients had cervical lymph node metastasis. Five out of 23 patients died: 3 patients had pulmonary metastasis and bone metastasis, and 2 patients died of other diseases. See Table 4 for details.

## Discussion

Local treatments are generally applied to preserve the vocal function and improve the quality of life of early glottic laryngeal carcinoma patients. Due to the local infiltration and growth of carcinoma and the advancement of treatment methods, the disease is characterized by a low probability of metastasis. Because EBRT is characterized by a lack of trauma, a good curative effect, and minimal effects on vocal function and quality of life, it is becoming more widely applied for patients with early glottic laryngeal cancer 12, 13. Because of its relatively non-invasive nature, good curative effect and minimal influence on vocal function and quality of life, TLM has progressively replaced the use of total laryngectomy/hemi-laryngectomy for early glottic laryngeal cancer patients 4, 7.

**Figure 4:**
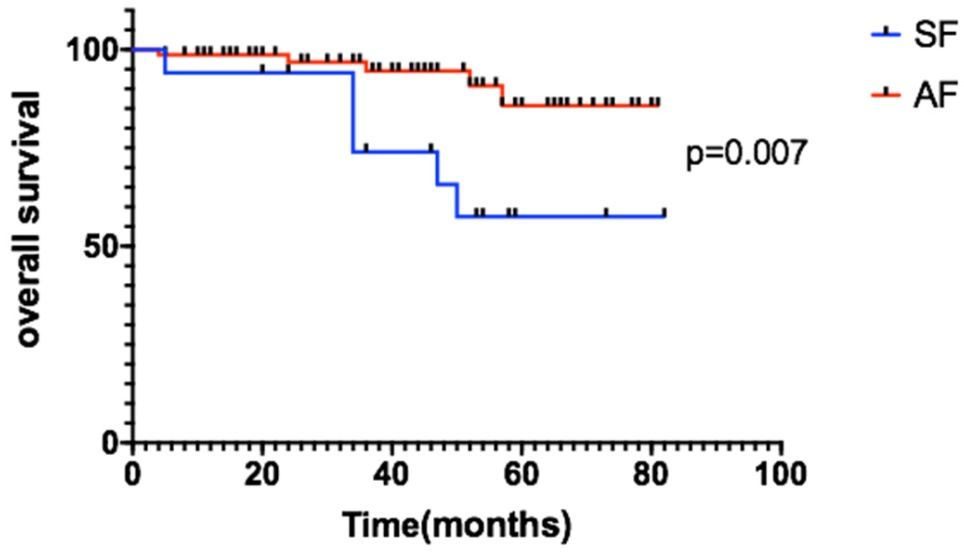
Patients with AF had longer OS than the SF group

This study demonstrates the use of EBRT and TLM in the treatment of stage T1-T2N0 early glottic laryngeal carcinoma. The total 3-year OS rates of the patients were 93.3% in the EBRT group and 95.4% in the TLM group, p=0.461. There was no significant difference between the two groups. The 3-year LC rates of the EBRT group and TLM group were 95.5% vs 89.2%, 89.2% vs 92.9% and 80% vs 82.9%, respectively.For subgroup analysis, we compare the baseline characteristics of EBRT and TLM groups, there were no significant difference between two groups (see details in table 2), and also the 3-year LC, PFS and OS of EBRT and TLM in the treatment of stage T1 or T2 showed no significant difference (see figure 2 for details). In the stage T1a, T1b and T2 subgroups, the 3-year OS rates were 93.6% vs 93.3%, 90.5% vs 95.9%, and 60% vs 64.2%, respectively. PFS in the stage T1a, T1b and T2 subgroups was 97.4% vs 89.2%, 89.2% vs 82.5% and 75% vs 82.9%, respectively. These results are similar to those reported in other studies 12, 13. Other studies also compared the efficacy of EBRT to that of TLM 7, 14. RJ De Santis et al. retrospectively analyzed data for early laryngeal cancer (Tis-T2) at Canada's largest head and neck hospital in 2006-2013 and analyzed 75 patients based on the initial treatment. The patients were divided into two groups: the radiotherapy group and the laser-ablation group. The 5-year DSF rates of the two groups were 90.8% and 93.3%, respectively, and there were no significant differences in the 5-year LC or PFS rate 2. A review in Martine 2017 described a comparison of treatments for T2 disease, and 48 studies showed no significant differences between radiotherapy (n=3191) and TLM (n=1156) at 5 years (75.81% vs 77.26%) 7. A phase I clinical study conducted by Leena-Maija Aaltonen et al. compared the efficacy of laser surgery (n=32) to that of EBRT (n=28) for patients with T1a glottic laryngeal carcinoma. The 2-year local recurrence rates were similar: 10% for the TLM group and 12% for the EBRT group 8. Thus, for patients with local early glottic laryngeal carcinoma (Tis-T2N0), EBRT appears to be effective, and the effect of radical radiotherapy is comparable to that of TLM treatment. New radiotherapy techniques, such as inverse intensity-modulated radiotherapy (IMRT), can increase the dose gradient of radiotherapy to reduce the OAR limit, even achieve similar plan quality to Cybernifes system 15. A number of retrospective studies, including studies by JCOG0701, Tosol Yu, Stokes, Stokes, and Tae Gyu Kim^1^, ^16^-^20^, have confirmed that compared with the use of SF radiotherapy, the use of AF radiotherapy for large segmentation therapy can increase the LC rate of early glottic laryngeal cancer and provide a survival benefit. The results showed that the application of IMRT combined with the large segmentation method for radiotherapy is reliable 21. Our study showed that the 3-year OS rates of the SF (n=17) group and the AF (n=74) group were 73.9% and 94.6%, respectively, p=0.007 (Figure 4). When acute and chronic side effects of the treatment were considered in this study, no grade 3 or higher toxic side effects were found, and there were no statistically significant differences between the radiotherapy technique groups 22. Most of the data have compared with the results of conventional radiotherapy and laser surgery. Due to the improvement of radiotherapy technology and segmentation mode, we believe that the treatment options of T1 stage patients will be further adjusted.

EBRT is not only effective in treating patients with early glottic laryngeal cancer but can maximize the preservation of the patient's vocal function 11, 23, 24. In this study, the VHI-30 scale was used to analyze patients before treatment, 6 months after treatment, and 12 months after treatment. VHI-30 is a widely used scale to evaluate voice, including 30 questions and three parts: function, physiology and psychology, studies have analyzed the match between VHI-30 and other multidimensional assessment, showing that the patient's vocal function could be successfully evaluated by VHI-30 9,11.Compared with the TLM group, the voice function of the EBRT group was well preserved. The vocal function of patients before treatment, 6 months after treatment and 12 months after treatment were analyzed by the VHI-30 scale. The vocal function scale scores of the EBRT group and the TLM group were 19.20 ±3.324 vs 21.65 ±9.809 (p=0.025), 10.24 ±6.093 vs 17.54±8.913 (p=0.001), and 9.45±5.112 vs 14.97 ±7.741 (p=0.001), respectively. In subgroup analysis, we separate the T1 and T2 stage patients to compare the vocal function after two different treatment, the baseline level of pathological stage of the patients showed no significant difference between two groups, for T1 stage, the vocal function scale scores at baseline, 6 months and 12 months after treatment of the EBRT group and the TLM group were 18.95±3.14 vs 17.94±7.43 (p=0.27), 9.92±7.84vs 16.05±7.56 (p=0.001), and 8.83±6.28 vs 12.43±6.82 (p=0.001), respectively. For T2 stage, the vocal function scale scores at baseline, 6 months and 12 months after treatment of the EBRT group and the TLM group were 21.69±3.39 vs 23.60±14.09 (p=0.37), 10.30±5.70 vs 17.93±11.84 (p=0.001), and 9.55±4.85 vs 15.07±10.15 (p=0.001), respectively. There was no significant difference in the baseline level between the EBRT and TLM groups, but there was a significant difference in vocal function between the EBRT group and TLM group at 6 months and 12 months after treatment. The vocal outcome results showed that the patient's vocal function was well preserved after EBRT treatment and that the vocal function at 6 months could predict the functional status within 1 year, which was similar to a previous report 10, 11. Relevant studies have also applied these scales to assess the effects of other treatments on vocal function 6. Compared to surgical treatment or TLM treatment, EBRT retains more complete vocal function 8, 14, with significant differences in VHI-F, VHI-P and VHI-E scores 9.

This study has some limitations. First, as a retrospective, single-center study, there may be selection bias. Second, the VHI-30 scale was used to evaluate the impact of voice function. This scale is based on patient self-evaluation rather than a related examination evaluation, which may lead to overestimation of the effects of vocal function. Third, there were more stage T2 stage patients in the TLM group than in the EBRT group, and the effects of TLM may have been underestimated. Despite these limitations, this is a large-scale population study exploring the efficacy of radiotherapy and its effects on voice function in patients with early glottic laryngeal cancer; it has high value as a reference and offers guidance in selecting treatment for patients with early glottic laryngeal cancer.

## Conclusion

EBRT is effective for patients with early glottic laryngeal cancer, offering a high LC rate and OS benefit with tolerable side effects. The use of the VHI-30 scale to evaluate the voice handicap of patients after EBRT for early glottic laryngeal cancer showed high vocal preservation.

## Figures and Tables

**Table 1:** Baseline characteristics

characteristics	EBRT group (n,%)	TLMgroup (n,%)	p value
gender			
male	84(92.3)	92(97.9)	0.079
female	7(7.7)	2(2.1)	
age			
<65	57(62.64)	63(67.02)	0.535
≥65	34(37.36)	31(32.98)	
smoking index			
<400	51(56.0)	44(46.8)	0.211
≥400	40(44.0)	50(53.2)	
T stage			
T1	78(85.7)	64(68.1)	0.004*
T1a	52(57.1)	41(43.6)	
T1b	26(28.6)	23(24.5)	
T2	13(14.3)	30(31.9)	
pathology			
H	60(65.9)	62(66.0)	0.443
M	23(25.3)	30(31.9)	
L	8(8.8)	2(2.1)	

EBRT=external beam radiation therapy; TLM=transoral laser microsurgery; T stage=tumor stage; pathology H=hyper-differentiation, M=middle-differentiation, L=less-differentiation

**Table 2:** Baseline characteristics of the T1 and T2 subgroup

	characteristic		EBRT (n,%)		TLM (n,%)		P value
T1 subgroup	gender	male	73	93.6	62	96.9	0.238
		female	5	6.4	2	3.1	
	age	<65	48	61.5	42	65.6	0.470
		≥65	30	38.5	22	34.4	
	smoking index	<400	45	57.7	34	53.1	0.267
		≥400	33	42.3	30	46.9	
	pathology	H	53	67.9	42	65.6	0.063
		M	18	23.1	21	32.8	
		L	7	9.0	1	1.6	
	T stage	T1a	52	66.7	41	64.0	0.860
		T1b	26	33.3	23	36.0	
	anterior joint involved	no	40	51.3	33	51.6	0.913
		yes	38	48.7	31	48.4	
T2 subgroup	gender	male	11	84.6	30	100.0	0.082
		female	2	15.4	0	0.0	
	age	<65	9	69.2	21	70.0	0.826
		≥65	4	30.8	9	30.0	
	smoking index	<400	6	46.2	10	33.3	0.621
		≥400	7	53.8	20	66.7	
	pathology	H	7	53.8	20	66.7	0.141
		M	5	38.5	9	30.0	
		L	1	7.7	1	3.3	
	anterior joint involved	no	0	0.0	4	13.3	0.198
		yes	13	100.0	26	86.7	

EBRT=external beam radiation therapy; TLM=transoral laser microsurgery; T stage=tumor stage; pathology H=hyper-differentiation, M=middle-differentiation, L=less-differentiation

**Table 3:** VHI-30 score at baseline and after EBRT or TLM

		EBRT			TLM		
		baseline (n=91)	6 months after EBRT (n=90)	12 months after EBRT (n=84)	Baseline (n=84)	6 months after TLM (n=80)	12 months after TLM (n=74)
Total	VHI F	7.78±2.62	4.24±3.326	4.02±2.966	12.71±6.4	13.95±6.068	12.23±5.58
	VHI P	7.11±1.66	3.59±2.203	3.48±1.746	5.73±1.99	2.92±0.960	2.32±0.662
	VHI E	4.31±1.082	2.41±1.931	2.06±1.819	4.30±1.079	2.76±0.845	2.20±0.741
	VHI total	19.20±3.324	10.24±6.093	9.45±5.112	21.65±9.805	17.54±8.913*	14.97±7.741*
T1 group	VHI F	7.58±2.35	4.19±2.87	4.25±4.73	9.16±5.16	9.77±6.08	8.22±5.87
	VHI P	7.05±1.66	3.74±2.23	3.00±1.63	4.27±0.87	3.02±0.96	2.42±0.69
	VHI E	4.32±1.06	2.36±1.87	1.58±1.55	4.24±1.07	2.93±0.83	2.37±0.76
	VHI total	18.95±3.14	9.92±7.84	8.83±6.28	17.94±7.43	16.05±7.56*	12.43±6.82*
T2 group	VHI F	14.00±3.62	4.54±5.18	3.99±2.53	20.88±7.28	14.71±7.98	14.13±6.89
	VHI P	4.46±1.65	2.69±1.68	3.56±1.74	4.04±1.10	3.04±0.98	2.50±0.76
	VHI E	4.23±1.19	2.69±2.16	2.14±1.84	4.58±1.15	2.67±0.80	2.21±0.71
	VHI total	21.69±3.39	10.30±5.70	9.55±4.85	23.60±14.09	17.93±11.84*	15.07±10.15*

VHI=voice handicap index, EBRT=external beam radiation therapy, VHI F=voice handicap index function, VHI P=voice handicap index physical, VHI E=voice handicap index emotion, *=p<0.05

**Table 4:** Failure pattern for patients treated with EBRT or TLM

	patients(n=185)			
	Total (n=185)	EBRT (n=91)	TLM (n=94)	p-value
Failure mode	No.(%)	No.(%)	No.(%)	
Local	18(9.73)	12(13.19)	6(6.38)	0.742
Regional lymph node	6(3.24)	3(3.30)	3(3.20)	0.503
Local and regional lymph node	2(1.10)	1(1.10)	1(1.10)	0.616
Distant	5(2.70)	4(4.40)	1(1.10)	0.425

EBRT=external beam radiation therapy, TLM=transoral laser microsurgery.
